# Effects of Chronic Stress and Comfort Food in Testicular Morphology in Adult Wistar Rats

**DOI:** 10.1590/S1677-5538.IBJU.2024.0515

**Published:** 2025-01-10

**Authors:** Carina F. Barnabé, Roger G. Marchon, Maria V. C. Pinto, Bianca M. Gregório, Anneliese Fortuna-Costa, Francisco J. B. Sampaio, Diogo B. De Souza

**Affiliations:** 1 Universidade do Estado do Rio de Janeiro – UERJ Unidade de Pesquisa Urogenital Rio de Janeiro RJ Brasil Unidade de Pesquisa Urogenital – Universidade do Estado do Rio de Janeiro – UERJ, Rio de Janeiro, RJ, Brasil

**Keywords:** Testis, Stress Disorders, Traumatic, Acute, Infertility

## Abstract

**Purpose:**

To investigate the effect of chronic stress on testicular morphology in adult Wistar rats, as well as the impact of comfort food consumption on these parameters.

**Material and methods:**

32 Wistar rats (10 weeks old) were divided into four groups: control (C), stressed (S), control + comfort food (C+CF), and stressed + comfort food (S+CF). Chronic stress was induced by the restraint method during 8 weeks in groups S and S+CF, while groups C and C+CF were maintained under normal conditions. Groups C and S received a standard rat chow diet, while groups C+CF and S+CF received both the standard chow and comfort food (Froot Loops®). After 8 weeks of experiment, all animals were euthanized and the testes were collected for histomorphometric, immunohistochemical and gene expression analysis.

**Results:**

Comfort food was preferred over standard chow in groups C+CF and S+CF, but this preference was more preeminent in stressed animals (S+CF). The consumption of comfort food resulted in testicular weight reduction. The seminipherous epithelium was reduced in group S in comparison to controls. While comfort food also reduced the epithelium in C+CF in comparison to controls, for group S+CF the comfort food ameliorated the stress-induced damage. The cell proliferation rate and the relative expression of StAR and BLC2 genes were similar between the groups.

**Conclusion:**

Both chronic stress and comfort food consumption resulted in morphological alterations of the testes but the consumption of comfort foods during chronic stress partially prevented the stress-induced detrimental effects on testes.

## INTRODUCTION

Stress is an imbalance disorder of the body, caused by external and internal factors (1). The exposure to those factors (stressors) directly and rapidly affects homeostasis, and when prolonged has a destructive effect on tissues, negatively influencing cell proliferation and differentiation, and prejudicing various body activities (2).

Chronic stress is associated with metabolic disorders and changes in energy homeostasis (1). Further, the high glucocorticoids levels contribute to the loss of control between appetite and satiety. This is related to an increased consumption of highly palatable foods (3-5). In turn, dietary change influences the response to chronic stress as, compensatory, it acts to inhibit some of the effects regulated by glucocorticoids (6, 7).

The consumption of comfort foods in society is closely linked to negative emotions and actions. That is, stressful factors (in a person's day or life) can trigger the search for comfort in food. This information is extremely relevant for stress studies since the consumption of comfort foods during stressful situations can act as a compensation for the situation itself, modulating the organic effects of stress (8).

It has been demonstrated that chronic stress promotes negative (morphological and functional) modifications in the testicles. Rats subjected to chronic stress stimuli showed impairment in sperm production and a reduction in serum testosterone levels, as well as a decrease in testicular weight (9). The apoptotic increase in testicular germ cells was seen after 7 days of (2-hour daily) immobilization stress (10). As testicles are androgen-dependent organs, the influence of stress on testosterone levels leads to several histological changes, which justifies the harm caused on sperm parameters (11, 12).

The resultant impact on the reproductive process is important. There is a clear association of stress, both acute and chronic, with male fertility. Further, the harmful effects of stressful stimuli can generate a vicious cycle of changes in reproduction; as the diagnosis of reproductive damage (caused by chronic stress), is per se a stressful event that increases the levels of stress, it compounds the reproductive condition (13).

The objective of this study is to investigate the effect of chronic stress on testicular morphology in adult Wistar rats, as well as the impact of comfort food consumption on these parameters. The present study raises three hypotheses to be answered: the first is that chronically stressed animals (more than non-stressed ones) increase the consumption of comfort foods; the second hypothesis is that chronic stress, despite the consumed diet, promotes deleterious effects on the testicle; finally, it is also hypothesized that the consumption of comfort foods can ameliorate the harmful effects of stress on the testicles.

## MATERIALS AND METHODS

### Animals

Thirty-two male Wistar rats were used in the experiments. All animals were bred in the Urogenital Research Unit animal facilities and were kept in a room with a controlled temperature (21°C ± 2°C) and artificial dark-light cycles (lights on from 7:00 am to 7:00 pm) and had free access to standard rat chow and water. This project was formally approved by the local ethics committee for the care and use of experimental animals (CEUA) under protocol number 004/2019 and followed national and international regulations on the experimental use of animals.

### Experimental design

When animals completed ten weeks of age they were randomly allocated into 4 groups as follows: Control group (C, n=8); Stressed group (S, n=8); Control + comfort food group (C+CF, n=8); and Stressed + comfort food group (S+CF, n=8).

For all animals, 50g of standard chow (Nuvilab CR-1, Quimtia, Colombo, Brazil) were offered daily. While animals of groups C and S received only standard rat chow, groups C+CF and S+CF received 30g of comfort food (Froot Loops, Kellogg Brazil, São Paulo, Brazil) in addition to the standard rat chow. Froot Loops was used as a comfort food due to its palatability, interesting aspect, and nutritional components.

Animals of groups S and S+CF were submitted to a chronic stress protocol by the immobilization method (14). Each animal was maintained in a rigid opaque plastic tube to restrain its movements, two hours daily, during eight weeks. Tubes with different diameters and lengths were adjusted weekly depending on the animal's size. All tubes had small holes to allow adequate ventilation. Meanwhile, the control groups (C and C+CF) were kept under normal conditions and not submitted to any stress procedure, though food was removed during the same period as that of stressed groups (2 hours daily) to avoid any bias in food intake measurements. All animals were killed the day after the last stress stimuli, when the animals were 18 weeks old.

### Dietetic, Biometric and glycemic analyses

For all groups, the standard chow present in each cage was weighted daily and completed for 50g per animal. For groups C+CF and S+CF, in addition to the standard chow, 30g of Froot Loops per rat was offered, and its consumption was also weighted daily. Food consumption (in grams and Kilocalories per body mass) as well as food preference (in percentage of consumption in grams; for groups C+CF and S+CF) was calculated and compared among groups. The food consumption was measured per cage (with 2 or 3 rats of the same group in each cage) and calculated individually (as a mean of the cage). Body mass was measured individually once a week.

Capillary blood glucose was measured (after a 12-hour fast) at the beginning (10 weeks of age) and at the end of the experiment (18 weeks of age) with a portable glucose monitor (Accu-Chek, Roche, São Paulo, Brazil).

### Morphometrical analyses

After eight weeks of experiments, the animals were submitted to euthanasia by isoflurane (Isofluorano, BioChimico, Itatiaia, Brazil). Both testes were dissected from their appendages, and their masses and volumes were measured by Scherle's method (15).

For morphometrical analyses, testes were fixed in Bouin solution by immersion for 24 hours, after which they were transversally sliced and immersed in buffered formaldehyde 3.7%. Furthermore, the samples were processed for paraffin embedding to obtain 5-µm-thick histological sections. Morphometrical analyses were performed in hematoxylin and eosin (H&E) stained sections using a microscope (BX51, Olympus, Tokyo, Japan) coupled with a digital camera (DP70, Olympus). All images were obtained and saved at a resolution of 2040 × 1536 pixels (16).

For each rat, the diameters of 125 cross-sections of seminiferous tubules were measured on images obtained at x100 magnification. The straight-line tool of the ImageJ software (National Institutes of Health, Bethesda, USA) was used to measure the tubule diameter. Only round-section tubules were considered, and the straight line always crossed through the center of the tubules. The seminiferous epithelium height was also measured in 125 tubules per rat by using images obtained at x200 magnification. For this analysis, 3 equidistant lines were drawn from the tunica propria of the seminiferous tubules to the last germinative cell, thus excluding the spermatozoa. The mean of these three lines was considered as the height of that seminiferous tubule (16).

The volumetric density (Vv) of each testicular structure was assessed using the point counting method (16). For each rat, 25 fields randomly captured were evaluated. Briefly, using the ImageJ software, a 99-point grid was superimposed over images at x400 magnification. However, to overlay the grid, the area of the image was previously measured using the "measure" tool of the ImageJ software. Each structure touched by the grid point was counted using the "cell counter" tool of ImageJ. The density was determined as a percentage of the analyzed field. In this way, we quantified the Vv of the tunica propria, seminiferous epithelium, tubular lumen, and intertubular compartment. The sum of the Vv[tunica propria], Vv[seminiferous epithelium], and Vv[tubular lumen] was considered the Vv[tubular compartment]. The points on the interstitial space were considered the Vv[intertubular compartment]. For each parameter, the result was expressed as a percentage and calculated from the average of the results of each analyzed image.

The cell proliferation rate was determined through PCNA (cell proliferation nuclear antigen) immunolabeled sections. Routine protocol for immunolabeling was performed, including the use of anti-PCNA primary antibody (clone PC10, cat. no. 13-3900, Life Technologies, Carlsbad, CA, USA), secondary antibody (MP-7452, Vector Laboratories, Newark, USA), diaminobenzidine revelation (SK-4105, Vector Laboratories, Newark, USA) and counterstaining with hematoxylin. The antigen retrieval was carried out in a water bath at 100ºC for 15 minutes, using citrate buffer at pH 6.0, and incubation with the primary antibody was carried out overnight at 4ºC. For each animal, 30 immunolabeled fields were captured at x400 magnification. The "cell counter" tool of ImageJ was used to count the labeled cells while the "free-hand selection" tool was used to determine the tubular area on each filed. The results were expressed as number of labeled cells per tubular squared millimeters.

### Gene expression analysis by reverse transcription quantitative real-time PCR (RT-qPCR)

Testis samples were collected from each animal for gene expression analyses by RT-qPCR. The samples were minced into small fragments, snap-frozen in liquid nitrogen and stored at −80ºC. Total RNA isolation was performed using the ReliaPrep™ RNA Tissue Miniprep System extraction kit (Promega, Wisconsin, USA), according to the manufacturer's protocol. Tissue RNA extraction was carried out using 2.8 mm diameter ceramic beads (CK28, Bertin Technologies, Montigny-le-Bretonneux, FRA) and the Precellys tissue homogenizer (Bertin Technologies), 2 cycles of 20 seconds at 5000 RPM. RNA purity and concentration were determined using the NanoDrop spectrophotometer (ND-1000 Spectrophotometer, Thermo Fisher Scientific, Massachusetts, USA).

Reverse transcription for the synthesis of complementary DNA (cDNA) and quantitative real-time PCR (qPCR) were performed with the GoTaq® Probe 1-Step RT-qPCR System kit (Promega), according to the manufacturer's instructions. The AriaMx Real-Time PCR equipment (Agilent Technologies, Santa Clara, USA) was used to carry out the experiment, and the resulting data were analyzed and quantified with the AriaMx Software. Each gene was evaluated in technical triplicate in all samples and normalized by the reference gene (GAPDH) using the formula: ${}^{\text{Δ}}\text{Ct}=\text{Ct (gene of interest) }-\text{Ct (reference gene) }$ . The ^Δ^Ct values of each group were used for statistical analysis (two-way ANOVA test). The relative fold change of gene expression was calculated using the 2−^ΔΔCt^ method (17). The qPCR primers were purchased from Thermo Fisher Scientific, with the following sequences (5’-3’): GAPDH Foward (GGT TAA AGT GGA AGG CGA TGT); GAPDH Reverse (CTC GCA TGC TGA TCA CAA TC); StAR Foward (CAC CAC CTT ACT TAG CAC TTC A); StAR Reverse (CAA GGA GAG GAA GCT ATG CAA); BCL2 Foward (CCA GGA GAA ATC AAA CAG AGG T); BCL2 Reverse (GAT GAC TGA GTA CCT GAA CCG).

### Statistical analysis

All parameter values were analyzed using the Kolmogorov-Smirnov normality test. Parametric data was compared by two-way ANOVA, with Tukey's post-test. Student's t-test was used to analyze food preference. All analyses were performed with the GraphPad Prism 9.0 software (GraphPad Software, San Diego, USA). Mean differences were considered significant when their p values were <0.05. All results are presented as mean ± standard deviation.

## RESULTS

### Dietetic, Biometric and glycemic analyses

Chronic stress did not alter the food consumption (measured in grams) among the groups. However, animals receiving comfort foods reduced the weight of food consumed by 9.5% (p=0.0004). Among the groups, S+CF had the lowest food consumption, 14.3% (p=0.0011) lower than group C, 10.2% (p=0.0324) lower than group C+CF, and 14.4% (p=0.0009) lower than group S. Also, two-way ANOVA showed that there is a significant interaction (p=0.0449) between the factors (stress and access to comfort food) regarding food consumption.

Also, chronic stress did not affect the food consumption measured by Kilocalories per body mass, but the access to comfort food did (p=0.0495). In this analysis, it was found that groups receiving comfort foods consumed 7.7% more calories than groups receiving only standard chow.

Animals that received standard chow and comfort food showed a significant preference for the latter, both in the C+CF and S+CF groups. The consumption of comfort food in the C+CF group was 73.3% higher than the consumption of standard chow (p<0.0001). For the S+CF group, this difference was in effect drastic: comfort food was consumed 123.5% more than standard chow (p<0.0001).

The initial body mass, the final body mass, the initial capillary blood glucose levels, and the final capillary blood glucose levels were similar among the groups, with no detected effect of stress, comfort food or the interaction among these parameters. The dietetic, biometric and glycemic data are presented in Table-1.

**Table 1:** Biometrical data of rats submitted to stress conditions with or without access to comfort foods.

Initial blood glucose (mg/dL)	89.25 ± 9.19	98.63 ± 13.21	95.38 ± 14.69		89.29 ± 11.88		0.7162	0.7221	0.0952
Final blood glucose (mg/dL)	89.19 ± 7.95	96.13 ± 13.67	99.71 ± 13.41		90.57 ± 8.88		0.7914	0.5520	0.0622
Initial body mass (g)	319.10 ± 57.85	348.00 ± 29.63	373.00 ± 41.24		313.40 ± 24.90		0.3125	0.6175	0.0618
Final body mass (g)	388.10 ± 70.45	378.60 ± 43.53	423.70 ± 38.93		364.90 ± 34.29		0.0648	0.5443	0.1759
Food intake (g)	27.77 ± 1.69	27.81 ± 1.95	26.51 ± 2.04		23.81 ± 1.67		0.0515	**0.0004**	0.0449
Food intake (kcal/kg BM)	0.24 ± 0.02	0.24 ± 0.02	0.27 ± 0.03		0.25 ± 0.03		0.1819	**0.0495**	0.1819
Food preference (g)			**SF**	**CF**	**SF**	**CF**	**p value** ^4^	**p value** ^5^	
			9.70 ± 0.95	16.81 ± 1.51	7.36 ± 1.69	16.45 ± 1.89	<0.0001	<0.0001	

C = Control group; S = Stress group; C+CF = Control + Comfort Food group; S+CF = Stress + Comfort Food group; SF = Standard Food; CF: Comfort Food; *p* value^1^ indicates stress effects; *p* value^2^ indicates comfort food effects; *p* value^3^ indicates interaction between the two factors; *p* value^4^ indicates the comparison of food preference of the C+CF group; *p* value^5^ indicates the comparison of food preference of the S+CF group.

Data expressed as mean ± standard deviation.

### Morphometrical analyses

The access to comfort food resulted in a reduction of 9.6% in the testicular mass (p=0.0309). Even so, there were no changes in testicular volume between animals receiving standard chow and those receiving comfort food. Also, the stress factor did not cause changes in testicular weight and volume.

**Figure 1:**
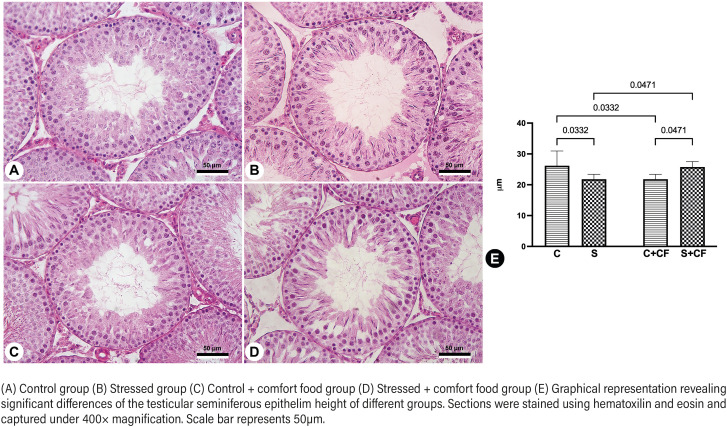
Photomicrographs representing the testicular seminiferous tubules of different groups.

The seminiferous tubule diameter and epithelium height were not affected either by stress or access to comfort food. Even so, two-way ANOVA showed that there was a significant interaction between these two factors (stress and comfort food). Tukey's post-test pointed out that group S had its epithelium height reduced by 16.7% (p=0.0332) in comparison to group C. Further, when comparing non stressed rats with (C+CF) or without access to comfort food (C), it was observed that comfort food promoted a reduction of 8.4% (p=0.0332) in epithelium height. On the other hand, comfort food had a different (and positive) effect in stressed animals: Group S+CF showed increased epithelium height by 18.2% (p=0.0471), in comparison to group S. Group S+CF also showed 7.5% higher values (p=0.0471) than those in group C+CF These results are illustrated in Figure-1.

(A) Control group (B) Stressed group (C) Control + comfort food group (D) Stressed + comfort food group (E) Graphical representation revealing significant differences of the testicular seminiferous epithelim height of different groups. Sections were stained using hematoxilin and eosin and captured under 400× magnification. Scale bar represents 50µm.

Stressed groups showed a 29.5% (p=0.0074) increase in tubular lumen Vv volumetric density in comparison to non-stressed animals. Post-test showed that groups S and S+CF had 60.3% (p=0.0122) and 56.4% (p=0.0201) higher tubular lumen Vv in comparison to group C. For the other volumetric densities studied, no differences were observed among the groups, as presented in Table-2. The analyses of the cell proliferation rate also showed a similarity between the experimental groups, without statistical differences among them.

**Table 2:** Testicular morphometric data of rats submitted to stress conditions with or without access to comfort foods.

	C	S	C+CF	S+CF	p value^1^	p value^2^	p value^3^
Testicular mass (g)	1.72 ± 0.12	1.75 ± 0.11	1.63 ± 0.19	1.51 ± 0.27	0.5314	**0.0309**	0.3054
Testicular volume (mL)	1.66 ± 0.18	1.64 ± 0.23	1.58 ± 0.07	1.48 ± 0.24	0.4755	0.1628	0.6476
Seminiferous tubule diameter (µm)	245.46 ± 8.81	237.04 ± 11.59	232.61 ± 10.37	238.51 ± 9.58	0.7714	0.1538	0.0862
Seminiferous epithelium height (µm)	26.17 ± 4.77	21.80 ± 1.58	23.97 ± 1.34	25.77 ± 1.74	0.2153	0.3896	**0.0005**
Seminiferous epithelium Vv (%)	53.47 ± 2.17	52.10 ± 4.88	49.84 ± 3.70	52.21 ± 5.30	0.7627	0.2969	0.2673
Tunica propria Vv (%)	3.68 ± 0.50	3.56 ± 1.00	3.73 ± 0.69	2.96 ± 0.44	0.1099	0.3147	0.2441
Tubular lumen Vv (%)	10.32 ± 2.34	16.54 ± 3.81	14.91 ± 1.74	16.14 ± 4.40	**0.0074**	0.1117	0.0611
Tubular compartment Vv (%)	69.17 ± 4.42	64.60 ± 19.39	68.50 ± 5.11	71.33 ± 9.39	0.8464	0.5012	0.4130
Intertubular compartment Vv (%)	30.45 ± 4.32	27.71 ± 7.17	30.91 ± 5.12	28.21 ± 9.22	0.3123	0.8562	0.9946
Nuclear proliferation rate (cells/mm²)	1268 ± 116.4	1278 ± 110.4	1255 ± 102.8	1311 ± 107.5	0.4404	0.8110	0.5974

C = Control group; S = Stress group; C+CF = Control + Comfort Food group; S+CF = Stress + Comfort Food group.

p value^1^ indicates stress effects; p value^2^ indicates comfort food effects; p value^3^ indicates interaction between the two factors.

data expressed as mean ± standard deviation.

### StAR and BCL2 gene expression analysis

The expression of the StAR gene was decreased by 26.45% in the stressed groups compared to the non-stressed animals, with fold change value of 0.54 of group S in relation to C, although the p-value did not reach statistical significance (p=0.0589). Furthermore, the post-test showed no differences among the four groups studied. The fold change of group C in relation to C+CF was 0.84. The fold change of group S in relation to S+CF was 1.07. The fold change of group C+CF in relation to S+CF was 0.68. Regarding the BCL2 gene, the relative expression levels were similar among the groups. The fold change of group C in relation to S was 0.97. The fold change of group C in relation to C+CF was 0.94. The fold change of group S in relation to S+CF was 0.91. The fold change of group C+CF in relation to S+CF was 0.95.

## DISCUSSION

This study sought to understand the effects caused by chronic stress on the testicles of adult Wistar rats, in addition to studying the relationship between stress and the eating behavior in this animal model. The activation of the hypothalamus-pituitary-adrenal axis by the stressor stimulus leads to endocrine dysregulation and elevation of glucocorticoids, which impacts many physiological pathways (1). The restraint stress methodology used in this study is associated with an increase in corticosterone levels and important impacts on the organism of rodents (6, 18).

Among the stress-induced physiological modifications, one explored in this study is the search for foods that stimulate comfort and could be a mitigating agent. In some cases, these mitigating agents can stop the progression or promote a recovery from the damages caused by stress (19). The selection of comfort food in this study was based on the sensory characteristics of rodents, as Froot Loops generates both visual, gustative, and tactile appeals. Comfort food was not chosen to study metabolic changes, but rather to analyze eating behavior during the chronic stress condition.

When comparing the numerical results regarding the food intake of all groups, the animals with access to comfort food reduced their food intake (measured in grams) but augmented the calories consumed, certainly due to the higher energetic density of the comfort food used. The effects of stress over food intake were not observed by our statistical methods, but the stress condition induced a marked modification of food preference on groups with access to comfort food. Both stressed and non-stressed groups preferred to eat comfort food than standard chow, as expected. However, this preference was more drastic for stressed animals: while non-stressed animals consumed 73.3% more comfort food than standard chow, stressed animals consumed 123.5%.

It is well known that changes arising from stress directly affect eating behavior, since satiety and appetite control are regulated by the hypothalamus, which is activated during chronic stress by raising the glucocorticoids bloodstream levels, impacting all hormonal regulation (3-5).

The preference for consuming comfort foods in non-stressed animals is explained by its palatability, but its higher preference by stressed animals can be associated with the search for pleasurable sensations as an amelioration of the stressful situations (4). Previous studies have pointed to the greater search for compensation in people and animals that have had negative mood stimuli. For these situations, the search for comforting sensations (including comfort food), is clearly a compensation for the mechanisms of the action of stress (8, 19).

The impact of chronic stress is not limited to physiological modifications. It also exerts great impact on the morphology of the organs, which is probably a consequence of the physiological modifications. The induced chronic stress in this experimental model was previously associated with morphological modifications in the urogenital system organs. Impacts on kidneys, bladder, prostate and penis were documented by this research group (12, 20, 21). The impacts of stress on testicular morphology and function were also explored before. In some studies, the impact of chronic stress over the testes was such that it caused a significant atrophy of the organ (12, 18). In the present study, such drastic changes were not observed. Testicular volume and weight were similar among the groups, suggesting that the chronic stress in this experiment was not as high as in previous studies. On the other hand, the access to comfort food reduced the testicular weight of the rats, which can only be explained by some alteration promoted by the altered diet, with higher levels of sugars (22).

Also, a decrease in the diameter of the seminiferous tubules in stressed animals was previously reported (9, 12), which was not observed in the present study, coinciding with no atrophy of the testicles. While the tubule diameter remained similar among groups, the seminiferous epithelium height was impacted by the interaction of stress and comfort food. The results of this analysis were very interesting; while both stress (group S) and comfort food (group C+CF) individually promoted a reduction in epithelium height, the stressed animals eating comfort food (group S+CF), showed better results. At first, this indicates that both stress and comfort food, alone, causes detrimental effects.

This alteration of seminiferous epithelium height may be associated with altered testicular function. As a result, from the germinative epithelium alterations, the production of spermatozoa will be affected. Such morphological changes may find an explanation in the decrease in steroidogenic activity. As spermatogenesis occurs in the seminiferous epithelium under appropriate hormonal conditions, dysregulations in testosterone levels can lead to alterations of this germinal epithelium (23). As is known, testosterone levels are commonly reduced during chronic stress by the interference of the hypothalamus-pituitary-adrenal axis on the hypothalamus-pituitary-gonadal axis (11, 12). The testicle is a highly proliferative and sensitive organ and depends on a very delicate microenvironment to properly produce hormones and masculine gametes, and any imbalance in homeostasis induces deleterious effects on its physiology (24). It is presumable that the impact of stress and comfort food consumption on the germinal epithelium may lead to altered sperm production, affecting the reproductive function.

Interestingly, when comparing the stressed animals (with and without access to comfort foods), a higher epithelium height of those with access to comfort food was observed. This suggests that comfort food acted as an ameliorating or preventing agent for the deleterious effects of chronic stress. This was actually so effective that the epithelium height of S+CF animals was similar to those of the control group. This reinforces the thesis that comfort food shows it is very beneficial during chronic exposure to stress, attenuating some detrimental effects normally caused by the stress condition.

While the analyses of seminiferous epithelium confirmed the hypothesis that comfort food may act as a stress mitigating agent, the data of tubular lumen does not. Tubular lumen was affected by stress similarly, whether in the groups receiving comfort food or those which did not. For this parameter, the access to comfort food neither prevented nor prejudicated the effects caused by stress.

The process of spermatogenesis depends on a delicate balance of apoptotic and proliferative factors. Previous work showed that chronic stress is capable of altering germ cells, impairing spermatogenesis (25) and reducing PCNA positive cells as well as altering PCNA gene expression (9). Despite these findings in the literature, our study did not observe any alteration regarding the proliferation rate. Regarding the apoptotic process, although there are studies that show genetic modulations arising from stress (26, 27), our data show no deregulation in BCL2 mRNA levels. BCL2 levels can provide good information due to its ability to inhibit induced apoptotic processes and act in balance with apoptosis-accelerating proteins, such as Bax (27).

The mechanisms of testosterone synthesis and regulation depend on several factors. Its bioconversion from the cleavage of cholesterol by the P450scc enzyme depends on adequate transport from the external mitochondria to the internal mitochondria, through the StAR protein. Studies prove that reproductive functionality and the synthesis of steroid hormones depend on good StAR gene expression, and regulated transcriptional and post-translational aspects (28). Endocrine changes arising from acute stress can lead to a reduction in the expression of StAR mRNA and as the stress period is prolonged, the stress adaptation mechanisms mediated by the HPA axis are able to resume their damage and increase gene expression (29). Although not supported by statistical analysis, the gene expression findings in our study may suggest that stress could lead to a repression of the StAR gene. It is worth noting that the p-value found was borderline, likely due to the variation in StAR gene expression between the study samples. In view of this, we believe that a long period of stress, as performed in our study, may indeed lead to a decrease in StAR gene expression.

Damage to the male reproductive function resulting from stress is studied scientifically, since this issue afflicts the lives of many men. Factors that can mitigate its possible harmful effects are important, due to the increase in stress exposure in today's society (7, 19). In this aspect, the present study is the first to show that the consume of comfort foods during stressful periods can ameliorate the harmful effects caused by chronic stress. Other novel finding is that the tendency for consuming comfort food is increased during stress. The data presented in this study support a better understanding of the mechanisms of action and the deleterious effects of stress and show that, for some parameters, the use of a comfort agent (even if this is a non-healthy food) can ameliorate the damage caused.

Possibly, the chronic stress imbalances the oxidant / anti-oxidant conditions in testicular tissue, as well as it happens in other testicular conditions (30). The increase in in reactive oxygen species, and/or decrease in antioxidant production may be a consequence of the chronic stress and a cause of the morphological alterations observed. Studies verifying the testicular oxidative stress damage during chronic stress, as well as studies focusing on the use of antioxidant agents for ameliorating the stress-induced alterations, are warranted.

As this is an animal study, one of the limitations is the direct transposing for human pathology. As it is known, it would not be practical to perform testicular biopsy in stressed patients for verifying the morphological condition. Even so, this manuscript adds important clue that can be used in clinical practice: a patient with infertility concern can be helped by (moderately) consuming comfort food during stressful situations. This is a non-harmful clinical recommendation that (relying on the present study findings) ameliorate the stress-induced testicular damages, improving the fertility.

The chronic stress methodology used in the study is different from the stressful stimuli that people receive in their lives. Further, as it is a repetitive method, some adaptation may occur, although the study results proved the stress condition. Further studies with other (non-repetitive) stress-induction methods are necessary to corroborate the results of this study. Nonetheless, these results help to understand some aspects of the impact of stress on testes morphology, its relationship with food preference, and the effects of comfort food consumption during stress conditions.

## CONCLUSIONS

The consume of comfort foods by chronic stressed rats partially prevented the stress-induced testicular morphological damage. This indicates that the use of comfort food during stressful situations can improve fertility. Also, the present study showed that induced chronic stress leads to an increased tendency to consume comfort food. Further, the study confirmed that chronic stress and the consumption of comfort foods resulted in morphological changes in the testes.
